# Analysis of health insurance data on dental treatment and the occurrence of osteoradionecrosis of the jaw

**DOI:** 10.1186/s12903-025-06114-y

**Published:** 2025-05-28

**Authors:** Ramona Schweyen, Stephanie Heinrich, Sara Lena Lückmann, Jeremias Hey, Steffen Fleischer

**Affiliations:** 1 Medical Faculty, Department of Prosthodontics, Martin Luther University Halle-Wittenberg, Halle (Saale), Germany; 2 AOK Sachsen-Anhalt, Magdeburg, Germany; 3 Medical Faculty, Institute of Health and Nursing Science, Martin Luther University Halle-Wittenberg, Halle (Saale), Germany; 4 Medical Faculty, Institute of Medical Epidemiology Biometrics and Informatics, Martin Luther University Halle-Wittenberg, Halle (Saale), Germany

**Keywords:** Head and neck cancer, Radiotherapy, Osteoradionecrosis, Health service research, Risk factors

## Abstract

**Background:**

Radiotherapy (RT) is a key component in the multimodal treatment approach for head and neck cancer (HNC). Post-therapeutic surgical and/or dental interventions on the jawbone carry a risk of developing osteoradionecrosis (ORN). To mitigate this risk, dental examinations and, if necessary, treatment should be conducted prior to RT. However, the consistent implementation of these recommendations in routine dental practice remains uncertain. This study aimed to evaluate whether insured persons of AOK Saxony-Anhalt (AOK ST) utilise dental services in accordance with current treatment recommendations and whether this behaviour influences the need for post-therapeutic tooth removal and the occurrence of ORN.

**Methods:**

Anonymised health claims data were analysed from individuals newly diagnosed with HNC between 2017 and 2021, who received RT and were continuously insured by AOK ST from 1 year before the start of RT to 2022. Three dependent variables were evaluated: dental treatment prior to RT, tooth extraction after RT, and ORN occurrence. Independent variables included sex, age, tooth extraction before RT, need for care, exemption from copayment, bisphosphonate prescription, diabetes, chronic obstructive pulmonary disease, alcohol abuse, chemotherapy, and guideline adherence.

**Results:**

Data from 1,086 patients with HNC diagnoses (75.9% male) were analysed. The median follow-up time from the first RT was 796 days (first quartile: 316 days; third quartile: 1,210 days). Twenty-one patients (1.9%) developed ORN after RT. More than 50% of the study population received dental care in accordance with guideline recommendations prior to RT. Need for care had the most significant negative effect on the utilisation of dental treatment prior to RT.

**Conclusion:**

This study did not find evidence of consistent implementation of the recommended guidelines for dental assessment/therapy prior to RT. Patients in need for care and those with chronic comorbidities were less likely to receive and/or require dental care. Although no significant influence on ORN development was observed, the reliability of this finding is limited by the small cohort size and low ORN incidence. Further studies with larger cohorts are needed to validate these findings.

## Background

Head and neck cancer (HNC) is the seventh most common cancer globally. Every year, more than 660,000 new cases and 325,000 deaths are registered [1, 2]. In many cases, radiotherapy (RT) plays a crucial role in multimodal tumour therapy approaches. However, high-energy radiation not only targets tumour tissue but also causes increasing fibrosis of the neighbouring tissue with reduced blood circulation, causing a lasting reduction in wound-healing capacity, particularly in bone [3]. Any post-tumour therapeutic intervention involving the jawbone therefore carries a risk of bone necrosis and the potential development of infected ORN [4]. This is especially true for patients whose tumour localisation results in increased irradiation of the alveolar bone or who underwent surgical resection of the jawbone during tumour surgery prior to RT [5].

In German-speaking countries, and in accordance with the current Arbeitsgemeinschaft der Wissenschaftlich-Medizinischen Fachgesellschaften (AWMF) guideline, the interdisciplinary treatment team for HNC also includes a dentist [6]. The dentist’s role is to determine whether dental treatment is required before initiating the planned head and neck RT to prevent subsequent infections originating from the roots of previously damaged teeth [7]. Following irradiation, decayed or severely periodontally damaged teeth can either promote the spread of oral pathogens into the vulnerable jawbone or, after their removal, result in significant wound-healing complications, potentially leading to ORN [8, 9].

To avoid this, acute dental pathological findings should be addressed, prognostically unfavourable teeth removed, and the patient instructed on the importance of meticulous oral hygiene before the start of RT. For this reason, based on the recommendation “Dental treatment of patients undergoing head and neck cancer radiotherapy” of the German Society for Dental and Oral Medicine, a dental examination should be scheduled approximately 6 to 2 weeks prior to the commencement of RT [10]. This examination should include an assessment of the dental and periodontal status, as well as the preparation of a current X-ray overview of the jaws to evaluate periapical bone conditions [10]. Teeth.

- with periodontal probing depth equal to or greater than 5 mm.
- with furcation involvement.
- with carious lesions reaching the pulp.
- which are impacted and retained.
- with large fillings, fractures, or significant occlusal wear.
- which are positioned in a high dose region of > 55 Gy.
- which were non-vital and without sufficient root canal filling.
- which were painful, sensitive to percussion, or revealed apical radiolucency.

should be extracted prior to radiotherapy. In addition, teeth, which are predestined to be severely affected by compromised mouth hygiene due to radiogenic trismus, should also be removed. Moreover, all dentulous patients should receive custom-made fluoride carriers of 5 mm-thick ethylene vinyl acetate and all patients should be encouraged to return to the dentist after RT [11].

Ultimately, the benefit of retaining each tooth, considering existing pre-damage and the anticipated effects of radiogenic xerostomia, must be weighed against its potential contribution to the development of ORN. Despite considerable advancements in RT technologies, selective tooth extraction is still often required [12]. From the clinician’s perspective, multimorbid patients and those suffering from stimulant abuse, as tobacco and alcohol, are more frequently subjected to the necessity of tooth removal [13]. However, evidence shows that, when the above explained dental treatment concept is followed, retaining teeth does not increase the risk of developing ORN [5].

However, most studies available in the literature have been conducted in study centres and university settings under defined interdisciplinary treatment conditions. It remains uncertain whether the recommended dental rehabilitation concept is consistently implemented in non-university settings, where logistical challenges may arise because of the more complex coordination required among various medical and dental treatment providers. This issue is particularly relevant for patients who may be restricted or fatigued by a long history of illness (e.g., chronic obstructive pulmonary disease [COPD] or diabetes) and/or who are in need for care.

The need for care refers to a condition in which a person with an illness or disability, often due to age, is no longer able to manage their everyday life independently and is therefore dependent on long-term care or help from others. People in need of care receive benefits from the German long-term care insurance system, which can take the form of financial benefits for informal support or formal support services.

Another factor negatively affecting the demand for dental services in relation to RT could be the perception that dental treatment entails undesirable additional costs for patients. Such barriers may lead to a significantly increased risk of late complications, such as ORN, which can sometimes have tragic consequences.

Unfortunately, gathering data on this patient group in non-university settings is challenging. Thus, the evaluation of health insurance data offers an opportunity to collect information on a large number of insured individuals treated at various locations and to assess the extent to which medical services were utilised within a given period.

The disadvantage of anonymised health insurance data is that although information on the type and period of a medical or dental treatment measure can be recorded, its concrete content can often not be specified. For example, it is possible to see in which quarter tumor resection and radiotherapy took place, but specific information on tumor stage or radiotherapy dose cannot be analysed. The same applies to the analysis of dental treatment measures - if dental billing items for a filling therapy are found in a patient’s health insurance data, this does not allow any conclusions to be drawn about the current tooth status or the extent of the carious lesions. In principle, however, conclusions can be drawn about the utilisation of medical and dental treatment measures.

In Germany, the general local health insurance company Allgemeine Ortskrankenkasse (AOK) is one of the largest statutory insurance providers. In Saxony-Anhalt, a German federal state with 2.2 million inhabitants, 40% of the population are insured by AOK Saxony-Anhalt (AOK ST).

The aim of this retrospective study was to determine whether insured persons of the AOK ST utilise dental services in the context of RT and whether this behaviour influences the need for post-therapeutic tooth removal and the occurrence of ORN. Additionally, we investigated whether there is an association between patients’ individual circumstances (e.g., age, sex, need for care, comorbidities such as diabetes and COPD, bisphosphonate intake, or additional chemotherapy) and the use of dental treatment in the context of RT, as well as its impact on the need for later tooth removal and the occurrence of ORN.

## Methods

### Study design

In this retrospective study, anonymised health claims data from individuals with a new diagnosis of HNC between 2017 and 2021, receiving RT, and continuously insured by AOK ST from 1 year before the start of RT until 2022 were analysed. Data from deceased patients were included up to the date of death. The available data from the health insurance company included sociodemographic data, billing data for primary care according to the uniform assessment standard [14], billing data for dental treatment according to the uniform assessment standard for dental services [15], and data for inpatient treatment with operation and procedure codes (OPS [16]), diagnoses using the International Classification of Disease German Modification (ICD-10-GM [17]),, and prescribed drugs classified using the anatomical therapeutic chemical classification (ATC) (Bundesinstitut für Arzneimittel und Medizinprodukte (BfArM) [18]).

Data were anonymised and provided by a data trust person of AOK ST. Raw data were imported, prepared, and checked by the authors following a previously established study protocol. The use of anonymised data fully complied with European and German federal law. Ethical approval was obtained from the Ethics Committee of the Faculty of Medicine, Martin Luther University Halle-Wittenberg (approval number: 2023-073). Because the analysis involved anonymised claims data, individual informed consent for participation in the study was not required. All the data were anonymised before analysis, and the results are presented in aggregate statistics only.

### Patients

Patients were included if they were insured with AOK ST throughout the study period, were diagnosed with a tumour in the head and neck region according to the ICD-10-GM classification (C00–C14 or C30–C32), and underwent RT. RT was identified when the OPS code (8–52) or billing data for outpatient irradiation using a linear accelerator or intracavitary/intraluminal brachytherapy (EBM 2532* or 2533*) appeared in the dataset. Patients were excluded from the analysis if they changed health insurer during the study period, were not insured continuously, or were not residing in Saxony-Anhalt.

### Data

The data included basic personal information, such as age, sex, year of tumour diagnosis, and date of death. Age was categorised into five groups (0–20, 21–40, 41–60, 61–80, and ≥ 81 years). HNC was defined based on diagnoses coded as C00–C14 or C30–C32 in the ICD-10-GM classification.

According to previous investigations, a higher risk for the occurrence of ORN due to the tumour localisation-related radiation field was assumed if ICD-10 diagnoses C01–C04, C06.2, or C14.8 were recorded [5]. Patients with ICD-10 diagnoses C00, C05, C06.0, C06.1, C06.8, C06.9, and C07–C14.2 were classified as having a lower ORN risk based on the tumour localisation-related irradiation field. Potential outcome-influencing factors recorded included comorbidities such as diabetes (E10–E14, ICD-10-GM), COPD (J44, ICD-10-GM), and alcohol abuse (F10, ICD-10-GM); need for care; and copayment exemption. Therapy-related factors recorded included bisphosphonates (M05B ATC) and additional chemotherapeutic agents (L01 ATC).

To assess dental examinations or restorations in relation to RT, any billed dental services associated with conservative or surgical treatment were analysed according to the Einheitlicher Bewertungsmaßstab für Zahnärztliche Leistungen (BEMA) (Table 1).

**Table 1:** List of billing items used to identify dental treatments in the context of tumour therapy

Dental therapy in relation to tumour therapy	BEMA / ICD-10 number	Time period for analysis
**Dental therapy before RT**		
Consultation before RT	BEMA: 01, 04, Ä1, Ä935d, Ä925a, Ä925b, Ä925d	2 quarters before start of RT (excluding quarter of RT)
Restorative intervention before RT	BEMA: 13a, 13b, 13c, 13d, 13dA, 13e, 13f, 13 g, 13 h, 105, 107, 31, 32, 34, 35, 43, 44, 45, Ä2381, 2382	180 days / 2 quarters before start of RT
Tooth extraction before RT	OPS: 5-230, 5-231 BEMA: 43–45	180 days / 2 quarters before start of RT
Bone surgery before RT	OPS 5-277.1-3 5-278.1-4 5-222.3,7 5-251.1 5-252.3,4 5-272.0-3 5-277.1-3 5-278.0-4 5-295.2,3 5-296.2,3 5-770.0,4,8,x 5-771.0-2 5-772.0-4,x, y	12 months before start of RT
Dental treatment prior to RT	Consultation before RT, restorative intervention before RT, or tooth extraction before RT	-
**Dental therapy after RT**		
Consultation within 90 days after RT	BEMA: 01, 04, Ä1, Ä935d, Ä925a, Ä925b, Ä925d	1 quarter after last RT
Consultation within 1 year after RT	BEMA: 01, 04, Ä1, Ä935d, Ä925a, Ä925b, Ä925d	1 year after last RT
Tooth extraction after RT	OPS: 5-230, 5-231 BEMA: 43–45	Complete study/observation period after last RT
**ORN after RT**	ICD-10: K10.2, T66	Complete study/observation period after last RT

### Statistical analysis

The anonymised data were descriptively analysed using absolute and relative frequencies for count data. Exploratory regression analysis was performed by fitting generalised linear models and calculating odds ratios (ORs) with 95% confidence intervals (CIs). Three dependent variables were analysed: dental treatment prior to RT, tooth extraction after RT, and ORN. Generalised linear models were fitted using three approaches: univariate models for all independent variables, a full additive model incorporating all independent variables, and a best-fit model using backward elimination.

The independent variables for tooth extraction and ORN as dependent variables were identical: sex (female/male), age (years), tooth extraction before RT (extraction/no extraction), need for care (need for care/no need for care), exemption from copayment (exemption/no exemption), bisphosphonate prescription (prescribed/not prescribed), diabetes (diabetes/no diabetes), COPD (COPD/no COPD), alcohol abuse (abuse/no abuse), additional chemotherapy (chemotherapy/no chemotherapy), and dental treatment prior to RT (yes/no). For ORN, two additional variables was included: ORN risk by tumour localisation (increased risk/normal risk) and by bone surgery during tumour resection prior to RT (bone surgery/no bone surgery).

Independent variables for dental treatment prior to RT were as follows: sex (female/male), age (years), need for care (need for care /no need for care), exemption from copayment (exemption/no exemption), diabetes (diabetes/no diabetes), COPD (COPD/no COPD), and alcohol abuse (abuse/no abuse).

## Results

### Description of the study cohort

The data provided by the data trust person of AOK ST included information from 1,086 patients with HNC diagnoses who underwent RT between 2017 and 2021 (male: *n* = 824, 75.9%). The median follow-up time from the first RT was 796 days, with a first quartile of 316 days and a third quartile of 1,210 days. During the observation period, which extended until the end of 2022, *n* = 546 (50.3%) patients died. Table 2 presents details on the age distribution, year of tumour diagnosis, comorbidities, social history, and therapy-associated factors within the study population. Nearly 60% of the study population was aged ≥ 60 years, and > 80% were exempt from copayment. Multiple tumour localisations represented the largest group, while tumours at the tongue base as a single localisation accounted for < 1% of the cases.

**Table 2:** Descriptive analysis of study cohort including age group, year of first tumour diagnosis, and comorbidities

**Age at diagnosis**										
Group	0–20 years		21–40 years			41–60 years		61–80 years		≥ 81 years
n (%)	1 (0.1)		9 (0.8)			447 (41.2)		528 (48.6)		101 (9.3)
**Year of first HNC diagnosis**										
Year	2017		2018			2019		2020		2021
n (%)	220 (20.3)		237 (21.8)			218 (20.1)		219 (20.2)		192 (17.7)
**Comorbidities**										**n (%)**
Diabetes										307 (28.3)
COPD										247 (22.7)
**Social factors**										**n (%)**
Alcohol abuse										369 (33.8)
Need for care dependency										425 (39.1)
Exemption from copayment										897 (82.6)
**Therapy-related factors**										**n (%)**
Tumour localisation	Naso-pharynx	Tonsil		Tongue base	Oral cavity		Cheek / parotid gland		Larynx, hypop-harynx	Multiple locations
ICD-10	C11, C30, C31	C09, C10		C01	C00, C02-C06, C08, C14		C07		C12, C13, C32	
n (%)	53 (4.9)	136 (12.5)		8 (0.7)	204 (18.8)		31 (2.9)		220 (20.3)	434 (40.0)
High risk for ORN according to tumour localisation and irradiation field										383 (35.3)
Bisphosphonate prescription										36 (3.3)
Chemotherapy treatment										604 (55.6)
Bone surgery before RT										318 (29.3)

Table 3 summarises the information on dental therapy in relation to RT. Treatment before the start of RT indicates that more than 50% of the study population received some form of dental care prior to RT. However, consultations with dentists after RT were considerably lower within the first 90 days, increasing to nearly 50% within 1 year after the last RT. Only 21 (1.9%) patients developed ORN following RT.

**Table 3:** Dental therapy in relation to RT and occurrence of ORN

**Dental treatment before RT (first RT)**	***n*** (%)
Consultation	575 (52.9)
Restorative intervention	331 (30.5)
Tooth extraction	214 (19.7)
Dental treatment prior to RT	591 (54.4)
**Dental treatment after RT (last RT)**	***n (%)***
Consultation by a dentist within 90 days	219 (20.2)
Consultation by a dentist within 1 year	479 (44.1)
Tooth extraction	61 (5.6)
**ORN after RT**	21 (1.9)

### Regression analyses

Possible factors influencing the implementation of dental treatment before the start of RT were analysed using univariate and multivariate approaches, as shown in Table 4. Only three variables remained in the best-fitting model: sex, need for care, and COPD. Among these, need for care had the most significant negative effect on the utilisation of dental treatment prior to RT (OR: 0.867; 95% CI: 0.816–0.92).

**Table 4:** Factors influencing utilisation of guideline-compliant dental treatment prior to RT

Variables	Univariate model OR (95% CI)	Full model OR (95% CI)	Best-fitting model OR (95% CI)
Age	0.999 (0.996–1.002)	0.999 (0.996–1.002)	NA
Sex (ref: male)	1.07 (0.998–1.146)	1.062 (0.99–1.139)	1.065 (0.995–1.14)
Need for care (ref: no need for care )	0.859 (0.809–0.912)	0.871 (0.819–0.926)	0.867 (0.816–0.92)
Exempt from copayment (ref: not exempt)	0.98 (0.906–1.06)	1.027 (0.948–1.111)	NA
Diabetes ref: (no diabetes)	1.023 (0.957–1.092)	1.025 (0.958–1.097)	NA
COPD (ref: no COPD)	0.889 (0.829–0.954)	0.908 (0.845–0.975)	0.905 (0.844–0.971)
Alcohol abuse (ref: no abuse)	0.933 (0.877–0.993)	0.957 (0.895–1.024)	NA
Bisphosphonate (ref: no bisphosphonate)	0.928 (0.787–1.095)	0.944 (0.8–1.115)	NA

NA: not applicable, ref: reference

Next, possible factors influencing the necessity of tooth removal after RT were analysed using univariate and multivariate approaches (Table 5). In the best-fitting model, only two variables remained: age and tooth extraction before RT, with tooth extraction before RT showing the strongest effect on tooth removal after RT (OR: 1.104; 95% CI: 1.067–1.143).

**Table 5:** Factors influencing need for tooth removal after RT

Variables	Univariate model OR (95% CI)	Full model OR (95% CI)	Best-fitting model OR (95% CI)
Age	0.997 (0.996–0.999)	0.998 (0.997–0.999)	0.998 (0.997–0.999)
Sex (ref: male)	1.006 (0.975–1.039)	1.013 (0.981–1.046)	NA
Dental treatment prior to RT (ref: no dental treatment prior to RT)	1.01 (0.983–1.039)	1.003 (0.975–1.031)	NA
Tooth extraction before RT (ref: no extraction)	1.117 (1.08–1.155)	1.104 (1.066–1.144)	1.104 (1.067–1.143)
Diabetes (ref: no diabetes)	0.985 (0.956–1.016)	0.998 (0.968–1.03)	NA
COPD (ref: no COPD)	0.985 (0.953–1.018)	0.995 (0.962–1.028)	NA
Alcohol abuse (ref: no abuse)	1.03 (1.001–1.06)	1.009 (0.978–1.041)	NA
Need for care (ref: no need for care)	1.004 (0.977–1.033)	1.006 (0.978–1.036)	NA
Exempt from copayment (ref: not exempt)	1.01 (0.975–1.048)	1.008 (0.971–1.045)	NA
Bisphosphonate prescription (ref: no prescription)	0.999 (0.926–1.079)	1.016 (0.942–1.097)	NA
Chemotherapy (ref: no chemotherapy)	1.012 (0.984–1.04)	0.985 (0.957–1.014)	NA

NA: not applicable, ref: reference

Finally, possible factors influencing the incidence of ORN were analysed using univariate and multivariate approaches (Table 6). In the best-fitting model, only three variables remained: dental treatment prior to RT, alcohol abuse, and need for care, all of which showed no clear or only very small effects on the incidence of ORN.

**Table 6:** Factors influencing incidence of ORN after RT

Variables	Univariate model OR (95% CI)	Full model OR (95% CI)	Best-fitting model OR (95% CI)
Age	1 (0.999–1.001)	1.001 (1–1.001)	NA
Sex (ref: male)	0.99 (0.971–1.009)	0.993 (0.973–1.012)	NA
Dental treatment prior to RT (ref: no dental treatment prior to RT)	1.017 (1.001–1.034)	1.014 (0.997–1.031)	1.017 (1–1.034)
Diabetes (ref: no diabetes)	0.996 (0.978–1.014)	0.998 (0.979–1.017)	NA
COPD (ref: no COPD)	1.006 (0.987–1.026)	1.008 (0.989–1.029)	NA
Alcohol abuse (ref: no abuse)	1.024 (1.007–1.042)	1.026 (1.007–1.045)	1.027 (1.009–1.045)
Need for care (ref: no need for care )	0.988 (0.971–1.004)	0.988 (0.971–1.006)	0.988 (0.971–1.004)
Bone surgery (ref: no bone surgery)	1.031 (1.013–1.05)	1.027 (1.007–1.048)	NA
Exempt from copayment (ref: not exempt)	0.998 (0.976–1.02)	0.993 (0.971–1.015)	NA
Bisphosphonate prescription (ref: no prescription)	0.98 (0.936–1.026)	0.99 (0.946–1.037)	NA
Chemotherapy (ref: no chemotherapy)	1.009 (0.992–1.025)	1.008 (0.99–1.026)	NA
High ORN risk due to tumour localisation and irradiation field (ref: no increased risk)	1.015 (0.997–1.032)	1.003 (0.984–1.021)	NA

NA: not applicable, ref: reference

## Discussion

Based on the available health insurance data, 54.4% of patients who underwent RT for a tumour in the head and neck region received dental examinations and/or treatments prior to the start of RT. This comparatively low rate of dental treatment did not result in an increased incidence of ORN during the follow-up period. At 1.9%, the incidence was at the lower end of the frequencies reported in the literature [4, 19, 20].

### Classification of the study cohort

The analysed data were obtained from a population of patients who were 75% male, with 50% aged between 41 and 60 years; this aligns with the average age and sex distribution observed in previous studies. These studies reported the highest disease rates in middle-aged men, despite an increasing proportion of female patients in recent years [21]. The median follow-up time from the first RT was 796 days, with the first quartile at 316 days and the third quartile at 1,210 days. By the end of the observation period in 2022, 50.3% of the patients had died, representing lower survival rates than the 5-year survival rates of > 50% described in the literature, depending on tumour stage and type [22, 23]. The comparatively high mortality rate during the observation period could be a reason for the rather low ORN incidence, as the risk of developing ORN increases with increasing follow-up time. Because the available data lacked information on tumour stage and type, these factors could not be considered for a more nuanced evaluation. However, based on the ICD-10-GM diagnoses, 35.2% of the patients were classified as having an increased risk of developing ORN due to tumour localisation and the associated probable radiation field. This classification was informed by previous studies using ICD-10-GM diagnoses [5]. Bone surgery during tumor therapy in the later irradiated jawbone, another known risk factor for ORN, affected 29.3% of the patients, consistent with previous findings [5, 24]. Additional chemotherapy and bisphosphonate use were also recorded. Chemotherapy is often associated with higher UICC stages or inoperable tumours and has been used as a surrogate marker for more advanced tumour findings in prior studies [5, 25]. Bisphosphonate-associated osteonecrosis of the jaws is well-documented and could potentially be misclassified as ORN in patients who also received RT [20].

Diabetes and COPD were recorded as comorbidities in 28% and 22.7% of the patients, respectively. These common diseases were registered in order to better classify the study cohort and cannot be used for a general medical assessment, for which it would have been necessary to record all co-morbidities. In this study cohort, the prevalence of these chronic conditions was significantly higher than in age-matched comparison groups in the literature, suggesting that most patients were already under regular medical care [26, 27]. Diabetes and COPD often indicate an increased need for dental care and might reflect a certain ‘experience of illness’, suggesting that patients accustomed to treatment regimens might be more likely to adhere to new ones [28, 29]. Otherwise, chronic illnesses increase the risk of developing fatigue making it difficult to follow new therapies. Alcohol abuse was recorded in 33.8% of the patients and is considered a major cause of tumours in the mucous membranes. However, as patients may provide socially desirable answers regarding alcohol consumption, the actual proportion could be higher [5]. Alcohol consumption has been shown to reduce patient compliance in various studies [30, 31], which justified its inclusion in the analysis. It would have been desirable to include smoking as a risk factor in the analysis. However, as this form of substance abuse is not coded by the health insurance funds, this aspect could not be included in the analysis and must remain the subject of clinical investigations.

The increasing proportion of people in need for care and/or patients with limited financial resources poses a growing challenge for the German healthcare system. This group of patients may face greater difficulties in accessing certain healthcare services [32, 33]. In Germany, patients requiring long-term care are assigned a ‘care degree’, which entitles them to additional assistance from health insurance companies and symbolises a certain level of need for support. In the present cohort, 39.1% of the patients were assigned a care degree. However, because of the limited number of participants, a differentiation into the five care degrees was not made, and a dichotomous classification into ‘need for care’ and ‘not need for care’ was used. To account for the financial background of the participants, the factor ‘co-payment’ was included in the analyses. In Germany, individuals below a certain income threshold are exempt from co-payments for healthcare services. This parameter was considered to evaluate whether patients exempt from co-payments were more likely to receive dental treatment prior to RT than those who were not exempt.

The analysis of guideline-compliant dental treatment prior to RT includes a dental examination with X-ray diagnostics and any necessary conservative and/or surgical interventions [10]. Ideally, these measures should be completed 2 weeks before the start of RT.

A major disadvantage of health insurance data is that the quality and scope of the dental and medical treatment provided cannot be taken into account. It could therefore be the case that, despite a billed examination, no guideline-compliant dental treatment was carried out before the RT because the patient did not want it, or that dental services were provided that were inadvertently not billed. Moreover, another challenge in the present study was that health insurance data could only be evaluated on a quarterly basis, while dentists are restricted to billing statutory health insurance for specific services within fixed periods. For instance, if a patient attended a regular dental check-up 4 months before RT, the dentist would not have been able to bill for a ‘dental examination’ again within 6 months, and either no billing or only a ‘dental consultation’ billing would have been recorded prior to RT. To provide a more realistic assessment, all dental billing items from the quarter of RT and the preceding quarter were classified as guideline-compliant therapy in this evaluation. Nevertheless, it is possible that not all dental treatment measures were captured. Patients who transitioned from inpatient care at an oral and maxillofacial surgery clinic to inpatient RT may have already undergone dental assessments and treatment during their initial stay, which would not appear as outpatient dental billing items. Furthermore, the dental status of patients was not accounted for. For example, edentulous and possibly frail patients may not have been expected to undergo additional dental examinations prior to RT.

Cases were classified as patients with the occurrence of ORN during the follow-up period if they had been assigned the diagnosis Inflammatory conditions of the jaws (K10.2) according to ICD-10-GM. It is important to note that this approach only captures diagnoses recorded in an inpatient setting because ICD-10 codes are rarely used in outpatient dental billing and documentation systems, where free-text entries are more commonly utilised. As a result, the actual number of ORN cases could be significantly higher than indicated by the available data.

### Factors influencing the implementation of dental treatment prior to RT

In the univariate analysis, the factors ‘sex’, ‘need for care’, ‘co-payment’, and ‘COPD’ were identified as influencing the likelihood of receiving dental treatment as part of RT. For instance, women were more likely to receive dental treatment before RT, whereas those requiring long-term care, those exempt from co-payment, and patients with COPD were less likely to do so. In the best-fit model, however, only ‘need for care’ and ‘COPD’ remained significant, with patients in need for care and those with COPD being 12% and 10% less likely, respectively, to receive dental treatment before RT. This may be attributable to a potentially reduced general condition in these patients.

The compliance of patients in need of care depends on both their condition of care dependency and the presence of a supportive environment [34]. For multimorbid, frail, and patients in need of care, seemingly non-urgent therapeutic measures often become a lower priority when faced with life-threatening diagnoses [35]. In the present study, no distinctions were made regarding care arrangements or settings of long-term care (e.g., formal vs. informal care, community vs. institutional care, or the level of care). Patients who had tumours with an additional diagnosis of COPD were less likely to receive dental treatment before RT than were patients without COPD. This may also reflect a generally poorer condition of these patients. In the future, efforts should be made to improve access to dental therapy for these patient groups, such as by arranging appointments directly between the radiotherapist and dentist.

### Factors influencing the removal of teeth after RT

In the univariate analysis of factors influencing tooth removal after RT, only age, 0.3% lower probability per year, and the variable ‘tooth removal before RT’, 12% higher probability if teeth had been removed before RT, were found to significantly affect the likelihood of tooth removal after RT. The multivariate best-fit model confirmed the significance of these variables.

It can be assumed that patients requiring tooth removal before RT likely had poor dental status or a critical tumour localisation, leading to a high probability of radiation caries due to increased radiation volume in the jaw and/or salivary glands. Clinically, the situation sometimes arises that prosthetically important abutment teeth for anchoring removable dentures, e.g. the canines, are left in place before RT despite clinically apparent risk factors. Retaining these teeth then enables the patient to continue wearing the extended dentures after RT. This proves to be psychologically important for patients during what is often a critical time. However, due to dry mouth and progressive radiation caries, the subsequent removal of further teeth may also be necessary in these cases. As a result, questionable teeth were removed before RT, and teeth damaged by radiation caries were removed after RT [12].

Regarding age, it is possible that critical teeth in older patients are less consistently extracted, with preference given to conservative preservation. This approach may reflect a focus on minimising invasive procedures for older, often frailer, and multimorbid patients [36].

### Factors influencing the occurrence of ORN after RT

With an overall comparatively low ORN incidence of 1.9%, the multivariate best-fit model identified an increased probability of developing post-therapeutic ORN associated with alcohol abuse (risk increase of 2.7%) and a slightly increased risk for the factor ‘dental treatment prior to RT’ (1.7%). This could partly be explained by the fact that patients already at increased risk before RT—due to factors such as tumour location or radiation volume—are likely to undergo more thorough dental examinations after RT, thereby increasing the likelihood of ORN diagnosis. Alternatively, this phenomenon may reflect limitations in the dataset, such as ORN cases not adequately coded as outpatient cases (see above) or patients who were excluded from additional dental treatment before RT due to known edentulism or a seamless transition from inpatient tumour therapy to inpatient RT without outpatient dental follow-up.

By contrast, a slightly reduced risk (1.2%) of developing ORN was observed in patients in need of care. This raises the question of whether ORN is diagnosed less frequently in patients requiring long-term care than in those not requiring such care, potentially because of reduced access to dental services. Another explanation could be a higher proportion of edentulous patients. Since the dental status was not recorded in the present study, this aspect cannot be explained in more detail.

Overall, the significance of the results presented here must be interpreted with caution due to the relatively small cohort, the short observation period and the poor survival rates. Furthermore, because the data consisted of anonymised health insurance records, treatment-specific details—such as tumour staging, tumour entity, radiation dose and volume, and dental status—that are recognised as relevant in the literature could not be included in the analysis.

## Conclusions

Contrary to the initial assumption, the present study did not find evidence of consistent implementation of the recommended guidelines for dental therapy prior to RT. Patients in need of care and those with chronic comorbidities were less likely to receive and/or require dental treatment before RT. Although no significant influence on the development of ORN was observed, the reliability of this result should be interpreted cautiously due to the relatively small study cohort and low incidence of ORN. Future studies with larger cohorts are necessary to further investigate and clarify the trends identified in this study, including comprehensive data on the type and location of the tumours including their interdisciplinary treatment, the dental status before and after RT, the type and manner of dental treatment in the course of tumour treatment as well as comprehensive information on the general condition of the patients.

## Data Availability

The datasets used and/or analysed during the current study are available from the corresponding author on reasonable request.

## Abbreviations

AOK ST: Allgemeine Ortskrankenkasse Saxony-Anhalt
AWMF: Arbeitsgemeinschaft der Wissenschaftlich-Medizinischen Fachgesellschaften
BEMA: Eineitlicher Bewertungsmaßstab für Zahnärztliche Leistungen
CI: Confidence interval
COPD: chronic obstructive pulmonary diesease
HNC: Head and neck cancer
ICD-10-GM: International Classification of Diseases 10 German Modification
OPS: Operation and procedure codes
OR: Odds ratio
ORN: Osteoradionecrosis
RT: Radiotherapy
